# Targeted Delivery of Mannosylated Nanoparticles Improve Prophylactic Efficacy of Immersion Vaccine against Fish Viral Disease

**DOI:** 10.3390/vaccines8010087

**Published:** 2020-02-15

**Authors:** Bin Zhu, Chen Zhang, Zhao Zhao, Gao-Xue Wang

**Affiliations:** Department of aquaculture, College of Animal Science and Technology, Northwest A&F University, Yangling 712100, China; bestzc@nwafu.edu.cn (C.Z.); zhaoz96122@nwafu.edu.cn (Z.Z.); wanggaoxue@126.com (G.-X.W.)

**Keywords:** targeted nanovaccine, mannose, carbon nanotubes, viral disease, immune response

## Abstract

Immersion vaccination is considered as the most effective method for juvenile fish in preventing viral disease, due to its convenience for mass vaccination and stress-free administration. However, immune responses following immersion vaccination are generally less robust and of shorter duration than those induced through intraperitoneal injection. Herein, to improve the efficacy of the immersion vaccine, we constructed a targeted single-walled carbon nanotubes-based immersion vaccine delivery system (CNTs-M-VP7), the surface of which are modified with mannose to allow antigen-presenting cells’ (APCs) targeting. The targeting ability of CNTs-M-VP7 was confirmed in vivo and in vitro. Critically, this immersion CNTs-M-VP7 vaccine could cross into the fish body through mucosal tissues (skin, gill, and intestine), and then present to immune-related tissues. Moreover, CNTs-M-VP7 could significantly induce the maturation and presenting process of APCs, which would then trigger robust immune responses. Altogether, this study demonstrates that the single-walled carbon nanotubes (SWCNTs)-based targeted nanovaccine delivery system shows the potential to be an effective prophylactic against fish viral disease.

## 1. Introduction

Viral disease outbreak is the most serious issue as it may cause severe losses to the economy in the aquaculture industry worldwide [1,2,3]. Vaccination is the most most effective strategy for fish viral disease prevention [4,5]. The route of administration is a vital factor influencing the efficacy and feasibility of vaccination. Injection, bath, and oral are the major administration routes used in aquaculture [6]. Injection is by far the most effective route, providing the highest protection. However, it is labor-intensive, causes handling stress to fish, and it is not feasible to vaccinate large numbers of juvenile fish via injection administration [7]. Oral and bath immunizations (mucosal routes) are the ideal way for fish at all life stages, especially the larval stage when fish are often the most susceptible to viral disease [8]. In addition, mucosal vaccinations are options superior to injection vaccination, given that mucosal vaccinations require lower cost and less handling stress. However, immune responses following mucosal vaccination are generally less robust, which is one of the main obstacles to overcome [9,10].

As the oldest living bony vertebrates, teleost fish possess both innate and adaptive immune responses [11,12]. The efficacious vaccines for teleost fish typically rely on the development of specific adaptive immune responses, as well as the innate immune responses, which are crucial in determining the subsequent adaptive response [13]. To enhance the efficacy of the mucosal vaccines, both of the specific immunity and innate immunity should be triggered. However, the innate responses need to be started at local mucosal tissues (skin, gill, and intestine) which are the barriers for mucosal vaccines [14,15]. Therefore, developments in efficient delivery technologies would be of great benefit to the application of mucosal vaccines.

Targeted delivery has been widely used in cancer treatment [16,17,18], but few studies focus on the prevention of fish viral disease. An efficient, targeted vaccine delivery system commonly consists of antigen, delivery carrier, and targeted ligand. The antigen is the key element that could induce the production of antibodies and arouse immune response of the host [19,20]. In this study, grass carp reovirus (GCRV), which has been recognized as the most pathogenic among all aquareovirus species [21,22], was studied as a model to evaluate the feasibility of targeted nanovaccine in preventing fish viral diseases. As the outer capsid protein of GCRV, VP7 is considered as a major antigen that could induce the host immune response [23]. Therefore, GCRV VP7 is the most common protein used in GCRV vaccine constructs. Single-walled carbon nanotubes (SWCNTs) have emerged as a promising candidate for the efficient delivery of biomolecules [24]. Upon covalent or noncovalent interaction, SWCNTs could be conjugated with antigens or drugs. This SWCNTs-based formulation has shown the intrinsic ability to cross biological membranes [25,26]. In recent years, SWCNTs have been widely used for vaccine delivery which could provide a significant means of enhancing and modulating immune response [27,28]. A targeted ligand is a vital element in a targeted delivery system [29]. Among various targeted ligands, mannose has been widely used for targeted nanovaccines, which can specifically recognize the mannose receptor on antigen-presenting cells (APCs) [30,31]. Therefore, mannose was modified and conjugated to antigens by chemical synthesis in this study.

Herein, we constructed a targeted immersion vaccine delivery system (CNTs-M-VP7). In this design, SWCNTs were loaded with antigen protein VP7 with mannose modification. CNTs-M-VP7 showed enhanced APCs uptake and maturation. The uptake kinetics of CNTs-M-VP7 was evaluated at the in vivo level. Moreover, strong immune responses in vaccinated fish were observed, demonstrates that CNTs-M-VP7 could act as an effective platform for prophylactic immersion vaccines against fish viral disease. Thus, the SWCNTs-based immersion vaccine with mannose modification provides a potential strategy against fish viral diseases.

## 2. Materials and Methods

### 2.1. Experimental Animal and Feeding Regime

Grass carps weighing 3.5 ± 0.5 g were provided from a GCRV-free farm in Guanzhou (Guangdong, China). Fish were bred under a 12/12 h (light/dark) regime at 28 ± 0.5 °C in a 300 L recirculating water tanks. Commercial dry feed pellets (Hellow Fish Dry Pellets; CVM Products, Beijing, China) were used to fed carps twice daily at a rate of 2% body weight. All procedures of animal experiments were approved by the guidelines of the Animal Experiment Committee, Northwest A&F University.

### 2.2. Cells

Grass carp ovary cell line (CO) were kindly provided by Zhejiang institute of freshwater fisheries (Huzhou, Zhejiang, China) and cultured at 28 ± 0.5 °C in humidified atmosphere with 5% CO_2_, and maintained in DMEM (Sigma, St. Louis, MO, USA) supplemented with 10% fetal bovine serum (ZETA LIFE, St. Louis, MO, USA); Grass carp kidney cells (CIK) cells (kindly provided by Prof. Ling-bing Zeng in Yangtze River Fisheries Research Institute, Wuhan, Hubei, China) were cultured at 25 ± 0.5 °C in humidified atmosphere with 5% CO_2_, and maintained in Medium 199 (Hyclone, St. Louis, MO, USA) supplemented with 10% fetal bovine serum; Grass carp macrophage was separated from grass carp head kidney by using a fish tissue mononuclear cell separation kit (Solarbio, Beijing, China).

### 2.3. Virus

The GCRV strain stored in our laboratory was cultured in CIK cells. The 50% tissue culture infective doses (TCID_50_) of the virus were performed according to established protocols [32].

### 2.4. Functionalized SWCNTs

Pristine SWCNTs were purchased from Chendu Organic Chemicals Co., Ltd., the Chinese Academy of Sciences (Chendu, China). The functionalized SWCNTs (o-SWCNTs) were prepared as described in our previous study [33], briefly, pristine SWCNTs were oxidized by H_2_SO_4_/HNO_3_ mixture (3:1, *v*/*v*) under reflux with stirring at room temperature for 48 h to form carboxyl groups on the surface of SWCNTs (o-SWCNTs).

### 2.5. Synthesis and Characterization of Targeted Nanovaccine

The polyethylenimine-functionalized SWCNTs (SWCNTs-PEI) were prepared according to a previous study [34]. Briefly, N-(3-dimethylaminopropyl)-N’-ethylcarbodiimide hydrochloride (EDC) and N-Hydroxysuccinimide (NHS) were introduced to a methanol suspension of o-SWCMTs, and then stirred gently at room temperature for 6 h. Then PEI was added to the mixture, followed by 24 h of stirring. Purified GCRV VP7 protein was prepared according to our previous study [33]. For conjugation of SWCNTs-PEI with VP7 antigen protein, SWCNTs-PEI (5.0 g) and butanedioic anhydride (5.0 g) were introduced in 150 mL of N, N-dimethylformamide (DMF) and ultrasonically dispersed at 45 KHz for 20 min, then the mixture was stirred for 36 h at 70 ℃. NHS (5.0 g) and diisopropylcarbodiimide (DIC; 10.0 mL) were added into the mixture and stirred for 24 h at room temperature. The resulting mixture was filtered and washed thoroughly with excess PBS (pH 7.2). The product (2.4 g) was ultrasonically dispersed in 500 mL PBS, and then the purified antigen protein (VP7) was gradually introduced into the suspension and stirred for 48 h at 4 °C. The mixture was filtered and thoroughly washed and then dried using a freezer-dryer (FD5-3, GOLD-SIM) to obtain SWCNTs based VP7 vaccine (CNTs-VP7). Unbound VP7 protein in the filters was determined using a bicinchoninic avid (BCA) protein assay kit (ComWin Biotech Co., Ltd., Beijing, China). To construct the SWCNTs-based mannosylated VP7 vaccine delivery system (CNTs-M-VP7), 1 g CNTs-VP7 was diluted in 100 mL PBS, then the solution was stirred for 2 h with (1,2-distearoyl-sn-sn-glycero-3-phosphoethanolamine-N-[methoxy(polyethyleneglycol)-3400]-Mannose) DSPE-PEG-Man (0.1 mg/mL).

For conjugation of fluorescein isothiocyanate (FITC), VP7/CNTs-VP7/CNTs-M-VP7 (1.0 g) was ultrasonically dispersed in PBS. FITC (0.5 g) was then introduced, and sonication continued for 3 h in the dark. The mixture was filtered and thoroughly washed and then dried using the freeze-dryer to obtain FITC-VP7/FITC-CNTs-VP7/FITC-CNTs-M-VP7.

The obtained constructs were characterized by the high-resolution TEM (HR-TEM; Tecnai G2 F20, Hillsboro, OR, USA), field emission scanning electron microscopy (FE-SEM, S-4800, Hitachi Ltd., Tokyo, Japan), dynamic light scattering (DLS) analysis (ZEN3600, Malvern, UK).

### 2.6. Safety Evaluation of the Constructed Vaccines

For the cytotoxicity of nanovaccines (VP7, CNTs-VP7, and CNTs-M-VP7), macrophages, CIK cells, and CO cells were seeded in 96-well plate at a density of 1 × 10^4^ cells per well and incubated overnight. A series concentration of nanovaccines was incubated with these three cells for 24 h, respectively. The relative cell viabilities were determined by the 3-(4,5-dimethyl-2-thiazolyl)-2,5-diphenyl-2H-tetrazolium bromide (MTT) assay (Sigma, St. Louis, MO, USA) following the standard protocol.

To evaluate the constructed nanovaccine in grass carp, 50 fish were bathed in CNTs-M-VP7 at 60 mg/L for 24 h. Then the treated fish were transferred to clean water and monitored daily. The health status of the treated fish was recorded for a period of 2 months post-vaccination.

### 2.7. Cellular and Tissular Uptake of Nanovaccine 

Fluorescence observation and flow cytometry were used to analyze the targeting ability of nanovaccines in vitro. The FITC-labelled nanovaccines (VP7, CNTs-VP7, and CNTs-M-VP7) (30 mg/L) were incubated with 7 × 10^5^ macrophages at 28 ℃ for 24 h, respectively. The unhandled macrophages were served as the control. After incubation, the cells were immunostained with the tissue-resident macrophage marker F4/80 primary antibody (1:250, Abcam, Cambridge, England), Cy3-labeled secondary antibody (1:1000, Beyotime, Beijing, China), and subsequently dyed with 1 mg/L 2-(4-Amidinophenyl)-6-indolecarbamidine dihydrochloride (DAPI) (Beyotime, Beijing, China). The confocal microscopy (Leica, Solms, Germany) was used to observe the fluorescence. For flow cytometry analysis, macrophages were seeded in 6-well plates and grown to a monolayer. The macrophages were incubated with the FITC labeled nanovaccines (VP7, CNTs-VP7, and CNTs-M-VP7) at the concentration of 30 μg/mL for 24 h, respectively. The treated macrophages were analyzed using BD FACSAria flow cytometry (BD, Franklin Lakes, NJ, USA) at 488 nm.

To evaluate the uptake of nanovaccines in vivo, grass carp were exposed to FITC-VP7, FITC-CNTs-VP7, and FITC-CNTs-M-VP7 at 30 mg/L for 6 h, respectively. Free FITC treatment was conducted as the control. Tissues, including kidney and spleen, were isolated and fixed in 4% paraformaldehyde. Then tissue sections were made and stained with 1 mg/L DAPI. After thoroughly washed with PBS, the tissues were observed in confocal microscopy (Leica, Solms, Germany). Image J software (NIH, Bethesda, MD, USA) was used to quantify the intensity of fluorescence in each group within the same region size.

### 2.8. APCs Activation Analysis

Grass carp were immersed with PBS, SWCNTs, Mannose, VP7, CNTs-VP7, and CNTs-M-VP7 at a concentration of 30 mg/L for 6 h, respectively. After the bath administration, fish were transferred to new tanks for breeding. At 1, 3, 7, and 14 days after the immersion, head kidney tissues were isolated from vaccinated fish in different groups, then those tissues were homogenized, centrifugated, and stored at −80 ℃.

The expression of cluster of differentiation 4 gene (*CD4*), cluster of differentiation 8 gene (*CD8*), major histocompatibility complex class I gene (*MHC*-I), major histocompatibility complex class II gene (*MHC*-II), interleukin 1β *gene* (*IL-1β*), and tumor necrosis factor α gene (*TNF-α*) were analyzed by quantitative real-time PCR. For RNA isolation and cDNA synthesis, total RNAs were obtained from the kidney tissue in each group (3 fish per group) at 1, 3, 7, and 14 days after vaccination with TRIzol reagent. HiScript Q Select RT SuperMix for aPCR (+ gDNA wiper) (Vazyme, Beijing, China) was performed to reverse transcribed the purified RNA into cDNA. Quantitative real-time PCR (qRT-PCR) was performed with CFX96 Real-Time PCR Detection System (Bio-Rad, Hercules, CA, USA) using AceQ^®^ qPCR SYBR^®^ Green Master Mix (Vazyme, Beijing, China) with the following procedure: 95 °C for 5 min and 40 cycles at 95 °C denaturation for 15 s, followed by 57 °C annealing for 60 s. The extracted DNA was used as a template for RT-PCR amplification with specific primers SM-F/R. The *18S* gene was used as an internal control (Table S1). All qRT-PCR reactions were performed for three biological replicates and repeated with two independent samples. Relative mRNA expression was calculated using the 2^−△△Ct^ method with the formula, F = 2^−△△Ct^, △△Ct = (Ct, target gene − Ct, reference gene) − (Ct, target gene − Ct, reference gene) control.

### 2.9. In Vivo Fluorescence Imaging

Grass carp were immersed with FITC-CNTs-M-VP7 at a concentration of 30 mg/L for 6 h. Then the treated fish were transferred to clean tanks for breeding. Tissues (gills, kidneys, spleen, liver, anterior intestine, middle intestine, and posterior intestine) were isolated from the vaccinated fish. Living body imaging system AniView 100 (BLT, Guangzhou, China) was used to observe vaccinated grass carp and their tissues (including gills, kidneys, spleen, liver, anterior intestine, middle intestine, and posterior intestine) at 5 different time points (0, 0.1, 6, 12, and 24 h) post-vaccination. The fluorescence images were analyzed by AniView 100 Living Imaging software (BLT, Guangzhou, China).

### 2.10. Vaccination

All vaccinations were performed at 28 °C. Disease-free grass carps (n = 1200) were used. Fish were randomly divided into 6 groups (200 fish per group): PBS, SWCNTs, mannose, VP7, CNTs-VP7, and CNTs-M-VP7. Each group was immersion vaccinated in 10 L nanovaccines (30 mg/L) for 6 h, respectively. After the vaccination, the fish in all groups were transferred to different tanks and monitored daily.

### 2.11. Serum Antibody Production, Enzyme Activities, and Virus Challenge

ELISA (Enzyme-linked immunosorbent assay) was used to analyze the antibody response and enzyme activity. For antibody production analysis, vaccinated fish (6 fish per group) were sampled weekly until 6 weeks. The unhandled grass carp were conducted as the control. The serum samples preparation and determination were performed as described in our previous study [33]. The purified recombinant VP7 protein (prepared in our lab) was used as the antigen. The rabbit anti-IgM polyclonal antibody, prepared in our lab, was used as the primary antibody. HRP-conjugated goat anti-rabbit IgG (Beijing CoWin Biotech Corp., Beijing, China) was used as the secondary antibody. These two antibodies were diluted 1:1000 with PBS containing 3% skimmed milk immediately before use. Tetramethylbenzidine (TMB) (Tiangen Biotech, Beijing, China) was used as a colorimetric substrate. The absorbance was measured at 450 nm using a precision microplate reader (Molecular Devices Corp., Palo Alto, CA, USA).

For enzyme activities, superoxide dismutase (SOD), complement, acid phosphatase, and alkaline phosphatase activities were measured using assay kits (Jiancheng Bioengineering Institute, Nanjing, China).

Challenge was performed on day 28 post-vaccination. Vaccinated grass carp (100 fish per group) were transferred to new tanks, and the water temperature was kept at 25 ± 1 °C. Each grass carp was injected intramuscularly with 30 μL of 3.0 × 10^4^ TCID50 of live GCRV in a saline buffer. Fish were observed daily for a period of 24 days post-challenge. Moribund fish were removed from the tanks and examined for clinical signs of GCRV. The relative percentage survival (RPS) was also calculated using Amend’s method [35].

## 3. Results and Discussion

### 3.1. Construction and Characterization of Targeted Delivery System

Functionalized SWCNTs are considered as promising vectors for peptide and protein antigens, due to their high loading capacity, biocompatibility, and needle-like structure [36,37]. For the construction of the current SWCNTs-based vaccines, vaccines are usually conjugated with SWCNTs by the amide condensation reaction. However, the water-dispersibility and biocompatibility still limit the use of SWCNTs as a vaccine carrier [38,39]. To tackle these obstacles, in the present study, SWCNTs were modified with polyetherimide (PEI) and then conjugated with antigen protein and functional polyethylene glycol-mannose. On the other hand, the dendritic PEI amines in SWCNTs are more favorable for further conjugating the desired functional groups and biomolecules. Importantly, there is a lack of specific targeting to APCs for the current SWCNTs-based vaccines. Antigen uptake by APCs is mediated by the mannose receptor which can bind to mannosylated polysaccharides or glycoproteins and then internalizes these ligands [40]. Numerous studies have shown that mannosylation of protein and peptide antigens could induce a significant enhancement of T cell response, resulting in stronger immune responses [41,42]. Therefore, we modified the nanovaccine with functionalized mannose.

**Figure 1:**
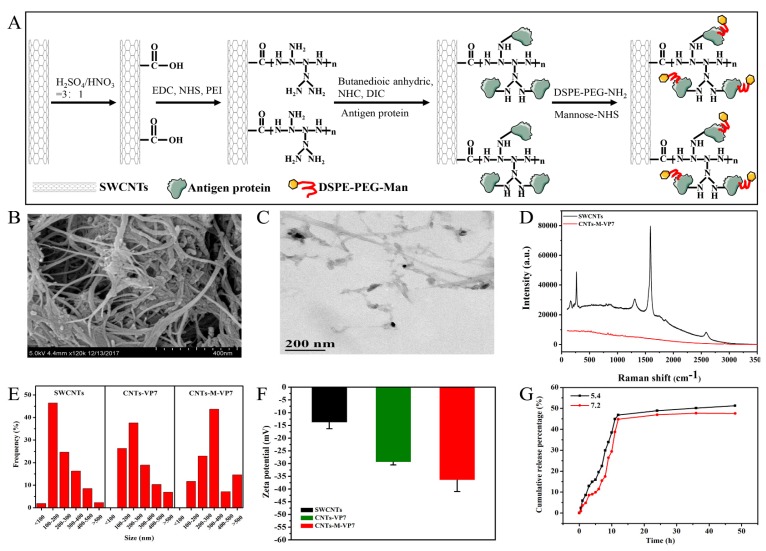
Characterization of nanovaccine. (**A**) Schematic illustration to show the step-by-step preparation of CNTs-M-VP7 nanovaccine. (**B**) Representative scanning electron microscopy image, and (**C**) transmission electron microscopy image of the SWCNTs-MG nanovaccine. (**D**) Raman analysis. (**E**) Dynamic light scattering analysis. (**F**) Zeta potential analysis. (**G**) Antigen release at 28 °C under two different pH conditions (pH 5.4 and 7.2) in vitro.

As illustrated in Figure 1A, we constructed an SWCNTs based mannosylated antigen delivery system (CNTs-M-VP7). Then the obtained nanovaccine was characterized. Scanning electron microscopy (SEM) and transmission electron microscopy (TEM) were used to analyze the morphology of CNTs-M-VP7, as shown in Figure 1B,C, the constructed vaccine is a tubular structure with mannosylated antigen proteins on its surface. Raman spectrum was used to confirm the obtained nanovaccine, as shown in Figure 1D, and two characteristic peaks of SWCNTs (G (1580 cm^−1^)) and D band (1303 cm^−1^)) were observed in SWCNTs. CNTs-M-VP7 only shows the characteristic peak of antigen proteins, which might due to the surface of SWCNTs being covered by antigen proteins. After conjugated with mannosylated proteins, the resulting SWCNTs showed increased size to be about 146 nm (Figure 1E), as well as a declining zeta potential of SWCNTs (Figure 1F). Bicinchoninic acid (BCA) protein assay and phenol-sulfuric acid colorimetry were used to quantify the content of antigen protein and mannose in the constructed CNTs-M-VP7, respectively. The results indicated the CNTs-M-VP7 nanovaccine contained 4.3% mannose and 48.34% antigen protein. We also analyzed the release behavior of antigen protein from CNTs-M-VP7 at 28 °C in a lysosomal environment (pH 5.4) and physiological environment (pH 7.4). As depicted in Figure 1G. A slightly faster release was observed in pH 5.4 than that in pH 7.2, and the cumulative release in 12 h achieved a high percentage (> 43%). These results above suggest the successful construction of a targeted delivery system (CNTs-M-VP7).

The safety of the constructed CNTs-M-VP7 nanovaccine was also analyzed in vitro and in vivo. A cell viability assay was used to evaluate the potential cytotoxicity of CNTs-M-VP7 toward macrophages, CIK cells, and grass carp ovary cell line (CO) cells. As depicted in Figure S1, there was no significant difference between the survival rates in all CNTs-M-VP7 treated cells with those in other treatments (PBS, o-SWCNTs, and mannose). For the safety evaluation in vivo, CNTs-M-VP7 vaccinated fish showed no lesion nor abnormality during a period of 2 months post-vaccination. Although the safety of SWCNTs remains controversial, by far, no conclusive evidence could verify the toxicity of SWCNTs. Numerous studies indicate SWCNTs are biocompatible for mammal and aquatic animals [39,43,44,45,46,47,48,49,50]. Moreover, the SWCNTs were modified by several active substances (PEI, antigen, mannose), which would greatly improve its biocompatibility [34]. The current work indicates that the SWCNTs-based mannosylated antigen delivery system could be used as the potential strategy for the construction of fish vaccines.

### 3.2. APCs Targeting Ability of the Constructed Nanovaccines In Vivo and In Vitro

**Figure 2:**
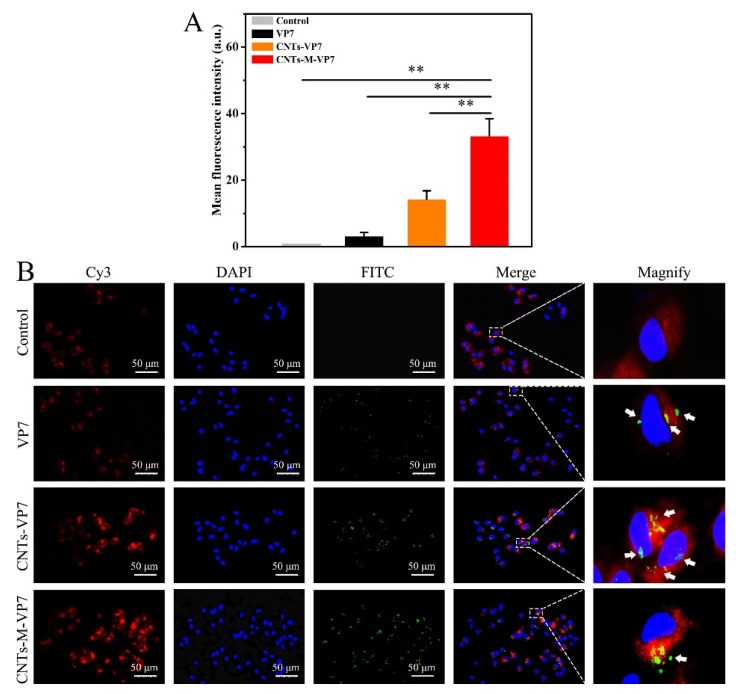
Cellular uptake of nanovaccine by macrophage in vitro. (**A**) Mean fluorescence intensity of cell uptake capability. Data are presented as the mean ± SD. *p* values were calculated by Student’s *t*-test (** *p* < 0.01, * *p* < 0.05). (**B**) Representative confocal microscopic images of macrophage incubated with VP7, CNTs-VP7, and CNTs-M-VP7, respectively. The unhandled macrophages served as the control. Vaccines were labeled with FITC (green channel), respectively; The cell nucleus was labeled with DAPI (blue channel); The mannose receptor was labeled with Cy3 (red channel).

Given the crucial roles of APCs in regulating immune responses, the APCs are considered as the attractive targets for prophylactic vaccines [51]. Herein, the APCs targeting ability of the constructed nanovaccines was evaluated in vitro and in vivo. For the in vitro evaluation, the internalization of the FITC-labeled nanovaccines (VP7, CNTs-VP7, and CNTs-M-VP7) was assessed using flow cytometry and confocal microscopy imaging. As shown in Figure 2A, the green fluorescence corresponded to the FITC-labeled nanovaccines. CNTs-VP7 loaded by SWCNTs and CNTs-M-VP7 loaded by SWCNTs and modified with mannose showed significantly enhanced cellular uptake by macrophages compared to the control group. Importantly, CNTs-M-VP7 showed significantly enhanced cellular uptake by macrophages compared with CNTs-VP7 without mannose modification. Similar results were also observed by fluorescence imaging, the macrophages incubated with CNTs-M-VP7 showed significantly higher green fluorescence than that treated with CNTs-VP7 (Figure 2B), indicating the stronger APCs targeting ability of CNTs-M-VP7.

**Figure 3:**
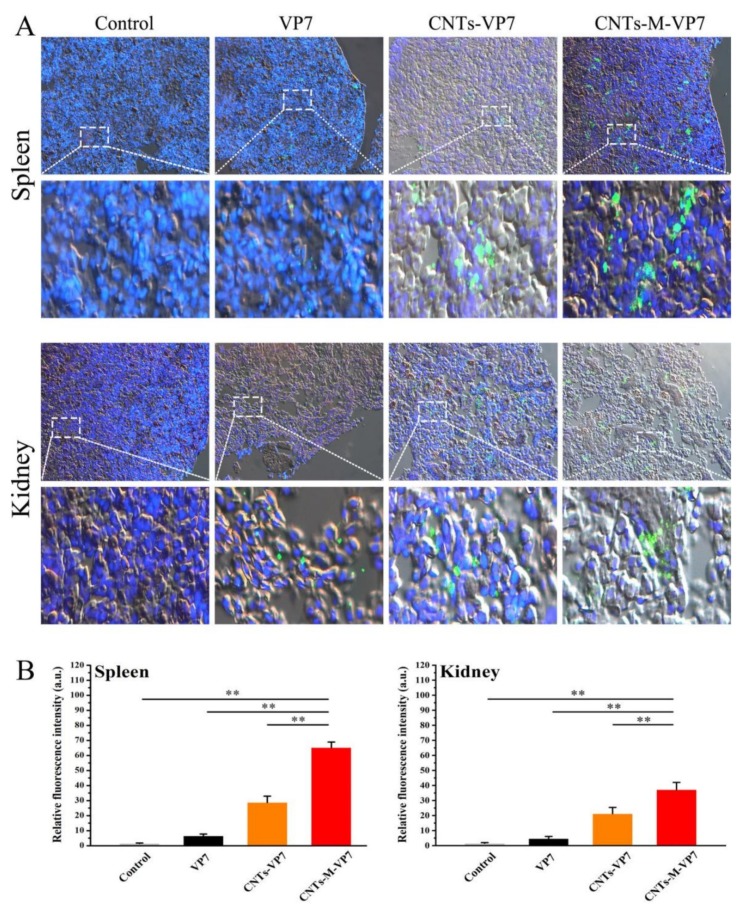
Uptake of nanovaccine in fish tissues. (**A**) The immunofluorescence images of fish tissues (spleen and kidney) after incubated with vaccines (VP7, CNTs-VP7, and CNTs-M-VP7), respectively. The vaccines were labeled with FITC (green channel); The cell nucleus was labeled with DAPI (blue channel). (**B**) Mean fluorescence intensity of vaccine in fish treated with VP7 (black column), CNTs-VP7 (Orange column), CNTs-M-VP7 (Red column). Free FITC treatment was conducted as the control (light grey column). Data are presented as the mean ± SD. *p* values were calculated by Student’s *t*-test (** *p* < 0.01, * *p* < 0.05).

We further analyzed the APCs’ targeting ability of CNTs-M-VP7 in vivo. Immunofluorescence results showed that no obvious green fluorescence was observed in the control group. On the contrary, both of the immune tissues (spleen and kidney) of fish that were vaccinated with CNTs-VP7 and CNTs-M-VP7 showed stronger green fluorescence compared with control and naked VP7 treated groups (Figure 3A). Furthermore, the relative fluorescence intensity in each group within the same region size was analyzed by Image J software to further confirm the in vivo uptake of nanovaccines. Similar to the fluorescence imaging results, the relative fluorescence intensity in CNTs-M-VP7 treated groups was significantly higher than that of VP7 and CNTs-VP7 treated groups (Figure 3B). As we know, fish kidney and spleen are the main immune organs containing abundant immune cells especially APCs [52,53]. The interactions between the nanovaccine and APCs would greatly determine the quality and quantity of induced immune responses. CNTs-M-VP7 displayed an enriched distribution in the kidney and spleen, indicating the enhanced immune response would be induced.

### 3.3. In Vivo APCs Activation by Nanovaccines 

**Figure 4:**
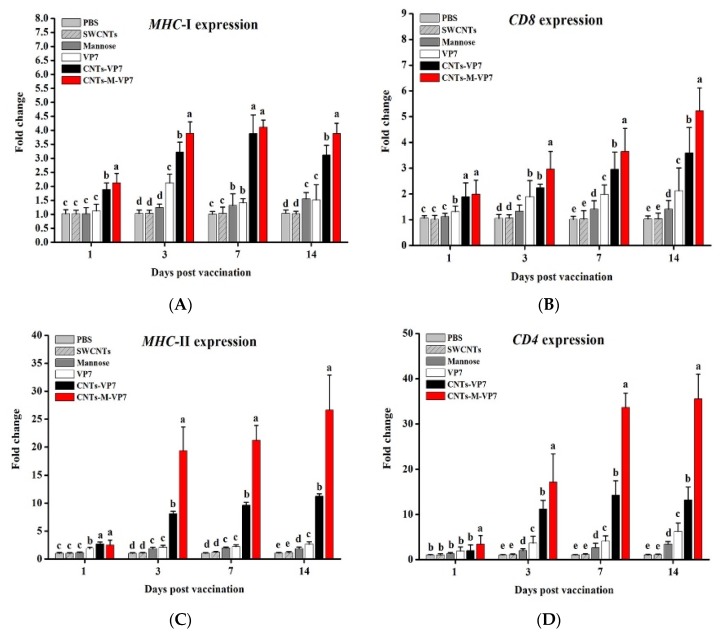

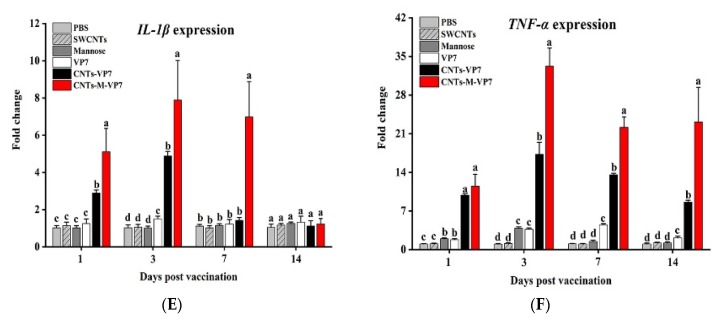
Expressions of (**A**) *MHC*-I, (**B**) *CD8*, (**C**) *MHC*-II, (**D**) *CD4*, (**E**) *IL-1β*, and (**F**) *TNF-α* in vaccinated fish. Data are means for three assays and represented as mean ± SD. *p* values were calculated by Duncan’s test. Data at the same sampling time with different letters (a, b, c, and d) are significantly different (*p* < 0.05).

The maturation of APCs is crucial to determining the quality and quantity of the subsequent immune responses. After presentation with antigens, immature APCs would be stimulated to mature states, which could activate T cells and then induce the immune response [54]. As the important factors participating in the antigen-presenting process, MHC-I, MHC-II, CD4, CD8, IL-1β, and TNF-α were used as the markers for mature APCs. Results showed that all these genes were significantly upregulated by the vaccination of CNts-VP7 and CNTs-M-VP7. Notably, at the same time point post-vaccination, the expression levels of these genes in the CNTs-M-VP7 group were significantly higher than those in the CNTs-VP7 group (Figure 4). CD4 and CD8 molecules are on the surface of T cells, which play an important role in the T cell-mediated immune responses by interacting with MHC molecules [55]. MHC class I could present antigens to activate T-killer cells, while MHC class II secreted on the surface of APCs could activate T-help cells and induce the subsequent immune response [56]. The higher expression levels of MHC-I, MHC-II, CD4, and CD8 molecules show the enhanced interactions between MHC molecules and CD molecules, indicating improved APCs activation. IL-1β and TNF-α, the typical pro-inflammatory cytokines, are highly relevant to antiviral immunity [57,58]. The increased expression levels of these two genes might reflect the stronger specific adaptive immune responses induced by CNTs-M-VP7. Therefore, the constructed CNTs-M-VP7 appeared to be highly effective in stimulating APCs activation and inducing the subsequent immune responses.

### 3.4. Delivery Kinetics of Targeted Nanovaccine

**Figure 5:**
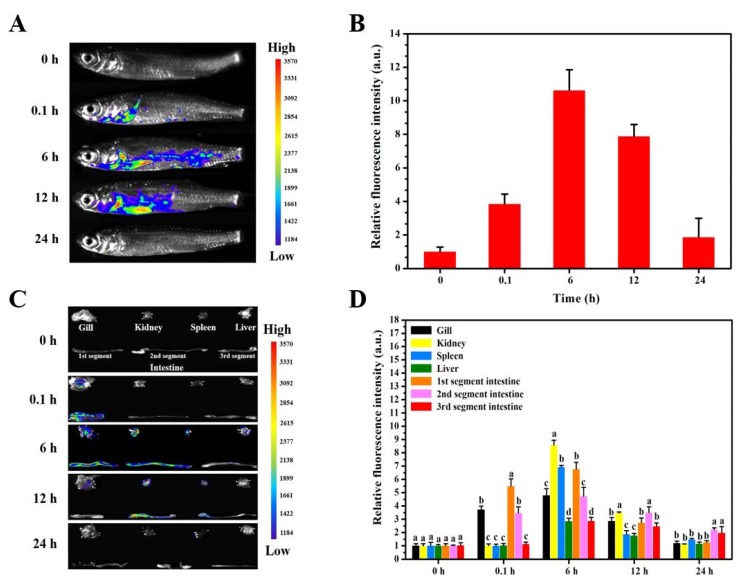
In vivo and ex vivo fluorescence images in vaccinated fish. (**A**) Representative in vivo fluorescence images for grass carp taken at different time points after vaccination; (**B**) Quantitative fluorescence signals of vaccinated fish; (**C**) Representative ex vivo fluorescence images of isolated fish tissues at five different time points; (**D**) Quantitative fluorescence signals of different fish tissues. Different colors in (A) and (C) represent the fluorescence intensity of FITC-CNTs-M-VP7 nanovaccine. The fluorescence intensity increases successively from purple to red. Data are means for three assays and represented as mean ± SD. *p* values were calculated by Duncan’s test. Data at the same sampling time with different letters (a, b, c, and d) are significantly different (*p* < 0.05).

Despite the targeting ability of CNTs-M-VP7 being confirmed in vitro and in vivo, its delivery kinetics remain unclear. Therefore, in vivo fluorescence imaging was used to track the FITC-labeled CNTs-M-VP7 in vaccinated fish. Grass carp were exposed to CNTs-M-VP7 at 30 mg/L for 6 h, then they were transferred to standard dilution water. The uptake of CNTs-M-VP7 in vaccinated fish was monitored at 0, 0.1, 6, 12, 24 h post-vaccination. As depicted in Figure 5, the uptake of CNTs-M-VP7 nanovaccine was gradually increased during a period of 6 h immersion vaccination, as expected, the uptake of CNTs-M-VP7 in gill and intestine was significantly higher than that in other organs during vaccination (*p* < 0.05). Notably, during this period of vaccination, the uptake of CNTs-M-VP7 in kidney and spleen was gradually increased, which corresponded with the results of CNTs-M-VP7 targeting ability in vivo. After transferred to standard dilution, the relative fluorescence intensity of CNTs-M-VP7 gradually declined. Interestingly, no appreciable fluorescence was observed in vaccinated fish at 24 h post the initial vaccination. The current results indicate that CNTs-M-VP7 could cross into the fish body through fish mucosal tissues including skin, gill, and intestine. Importantly, CNTs-M-VP7 might be migrated to immune organs (spleen and kidney). However, the real delivery kinetics of CNTs-M-VP7 requires further investigation.

### 3.5. Immune Responses Induced by CNTs-M-VP7 Nanovaccine

**Figure 6:**
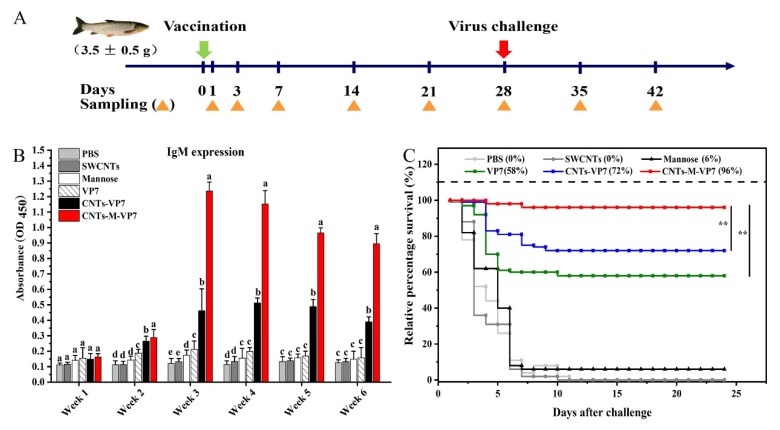
Immune responses in vaccinated fish. (**A**) Schematic overview of the vaccination/challenge protocol and sampling time point. (**B**) Serum specific antibody levels of fish vaccinated with different vaccines. Data are presented as mean ± SD (n = 6) and analyzed with Duncan’s test. Data at the same sampling time with different letters (a, b, c, and d) are significantly different (*p* < 0.05). (**C**) Relative percentage survival after artificial challenging with GCRV in vaccinated grass carp (n = 100, per group). The survival percentage was recorded daily and calculated at the end of the monitored period. *p* values were calculated by Log–rank (Mantel–Cox) Test (* *p* < 0.05, ** *p* < 0.01).

Immune responses in vaccinated fish were analyzed. Most efficacious vaccines can induce high levels of specific antibody production that is crucial in determining the quality of the subsequent adaptive immune response. Therefore, first, we analyzed the serum IgM production in vaccinated fish. As shown in Figure 6B, CNTs-VP7 and CNTs-M-VP7 showed significantly increased secretion levels of serum IgM production and reached a maximum in the third-week post-vaccination. Notably, CNTs-M-VP7 stimulated higher levels of serum IgM production than CNTs-VP7 at each of the examined time points. In addition, no appreciable IgM expression was detected in the PBS and SWCMTs groups during 6 weeks post-vaccination. Analogous to mammalian, teleost IgM contributes predominantly to systemic immunity [11,12]. The enhanced serum IgM production indicates that CNTs-M-VP7 could elicit a robust systemic immune response in vaccinated fish. Given the crucial role of enzyme activities in regulating host immune responses [59,60,61], we also analyzed these above enzyme activities including complement, superoxide dismutase (SOD), acid phosphatase (ACP), and alkaline phosphatase (AKP) activities in vaccinated fish. These enzyme activities were significantly induced by CNTs-M-VP7 (Figure S2), consistent with the serum IgM production results.

To further evaluate the prophylactic effects of CNTs-M-VP7 nanovaccine, GCRV disease was used as a model to evaluate the feasibility of CNTs-M-VP7 nanovaccine in the prevention of fish viral disease. As shown in Figure 6C, all fish in the PBS and SWCNTs groups were infected with GCRV with 100% cumulative mortality. The survival rate of fish treated with CNTs-M-VP7 was 96%, whereas it was 58% and 72% for VP7 and CNTs-VP7 groups, respectively. Those results strongly evidence that our developed SWCNTs-based immersion vaccine with mannose modification could be a rather attractive strategy for the prevention of fish viral diseases.

## 4. Conclusions

In general, we have constructed an efficient immersion vaccine delivery system (CNTs-M-VP7) consisting of SWCNTs as the vaccine carrier, GCRV VP7 protein as the antigen, and mannose as the APCs-targeting moiety. The targeting ability of CNTs-M-VP7 was confirmed in vivo and in vitro. Moreover, CNTs-M-VP7 could enter into the fish body and present to immune-related tissues via immersion administration, resulting in the robust immune response of the host. This work provides an outlook for future vaccination strategies against fish viral diseases.

## Supplementary Materials

The following are available online at https://www.mdpi.com/2076-393X/8/1/87/s1, Figure S1: Safety evaluation of nanovaccine in vitro. Relative cell viability of (A) grass carp macrophage, CIK cells (B), and CO cells (C) after incubation with different concentrations of VP7, CNTs-VP7, and CNTs-M-VP7 for 24 h. Values are presented as as mean ± SD, Figure S2. Enzyme activities in vaccinated grass carp: (A) Superoxide dismutase (SOD) activity; (B) Complement activity; (C) Acid phosphatase (ACP) activity; (D) Alkaline phosphatase (AKP) activity. Data are means for three assays and represented as mean ± SD. *p* values were calculated by Duncan’s test. Data at the same sampling time with different letters are significantly different (*p* < 0.05), Table S1: Primers used for the analysis of mRNA expression.
