# Supplementary material for: Targeted Delivery of Mannosylated Nanoparticles Improve Prophylactic Efficacy of Immersion Vaccine against Fish Viral Disease
Source: Vaccines (Basel). 2020 Feb 15;8(1):87. doi: 10.3390/vaccines8010087 (PMC7157632; doi:10.3390/vaccines8010087)
Supplement: Supplementary file 1 [file vaccines-08-00087-s001.pdf]

vaccines

MDPI

Article

Targeted Delivery of Mannosylated Nanoparticles Improve Prophylactic Efficacy of Immersion Vaccine against Fish Viral Disease

Table S1. Primers used for the analysis of mRNA expression.

Genes	Accession no.		Primer Sequences (from 5′ to 3′)	Product Size (bp)
18S	EU047719	Forward	ATTTCCGACACGGAGAGG	90
		Reverse	CATGGGTTTAGGATACGCTC	
MHC-I	AY391782	Forward	CCTGGCAGAAAAATGGACAAG	271
		Reverse	CCAACAACACCAATGACAATC	
CD4	KX033448.1	Forward	GTGCAGAGCTGCACTGCGACA	240
		Reverse	GCACTATTTTGCCTCCTTCAGA	
MHC-II	EF140725.1	Forward	GAAGGAGAAATTACTCTA	138
		Reverse	TTTCTCTTGTGGATATTTGTAA	
CD8	GQ355586	Forward	GAGTCTCTGCACGGATCTAT	172
		Reverse	GTGTAGTGTTCCGAATTTAAGT	
IL-1β	EU047716	Forward	GGAGAATGTGATCGAAGAGCGT	448
		Reverse	GCTGATAAACCATCCGGGA	
TNF-α	EU047718	Forward	TGTGCCGCCGCTGTCTGCTTCACGCT	291
		Reverse	GATGAGGAAAGACACCTGGCTGTAGA	

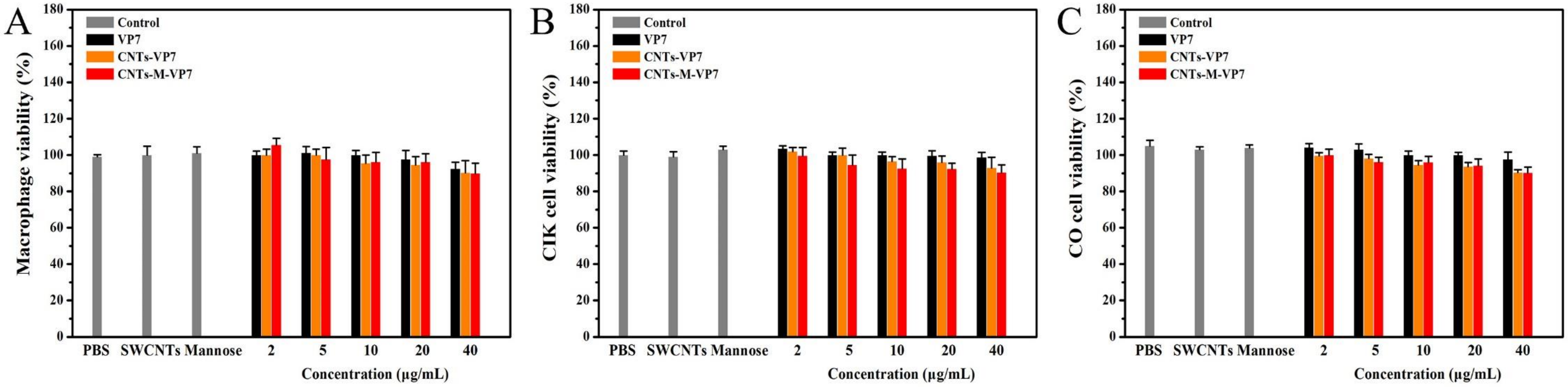

Figure S1. Safety evaluation of nanovaccine *in vitro*. Relative cell viability of (A) grass carp macrophage, CIK cells (B), and CO cells (C)after incubation with different concentrations of VP7, CNTs-VP7, and CNTs-M-VP7 for 24 h. Values are presented as as mean ± SD.

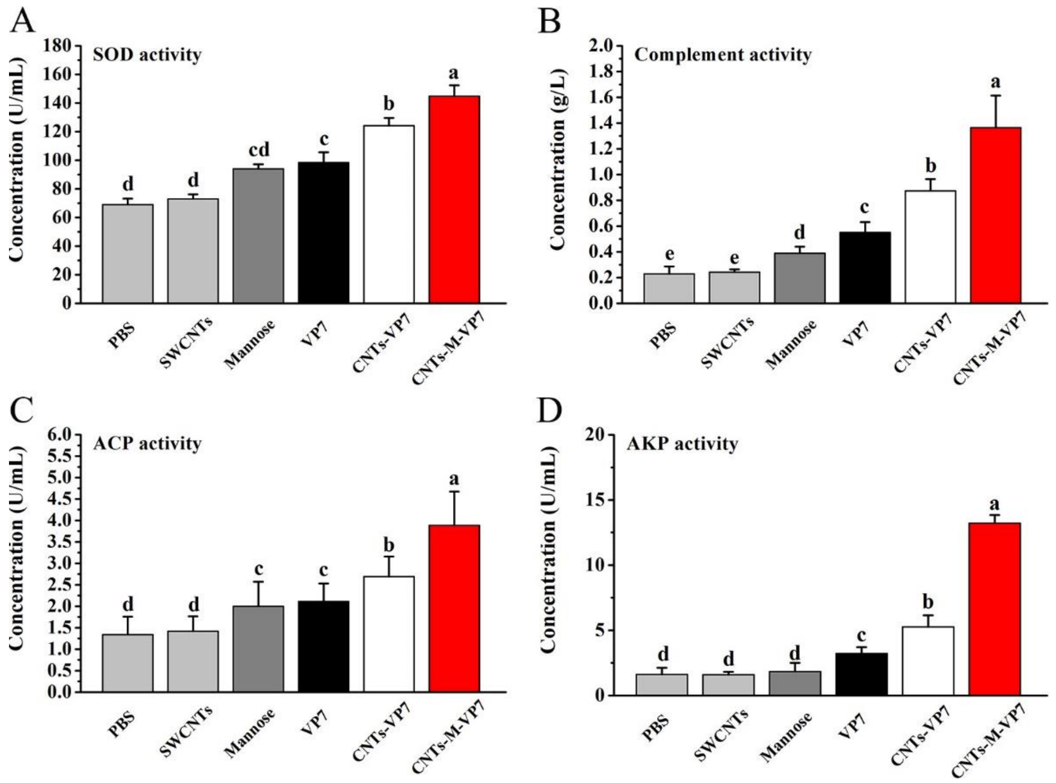

Figure S2. Enzyme activities in vaccinated grass carp: (**A**) Superoxide dismutase (SOD) activity; (**B**) Complement activity; (**C**) Acid phosphatase (ACP) activity; (**D**) Alkaline phosphatase (AKP) activity. Data are means for three assays and represented as mean ± SD. *p* values were calculated by Duncan's test. Data at the same sampling time with different letters are significantly different ($p < 0.05$).

© 2020 by the authors. Submitted for possible open access publication under the terms and conditions of the Creative Commons Attribution (CC BY) license (http://creativecommons.org/licenses/by/4.0/).
